# Use of Echocardiography and Heart Failure In-Hospital Mortality from Registry Data in Japan

**DOI:** 10.3390/jcdd8100124

**Published:** 2021-09-30

**Authors:** Kenya Kusunose, Yuichiro Okushi, Yoshihiro Okayama, Robert Zheng, Michikazu Nakai, Yoko Sumita, Takayuki Ise, Koji Yamaguchi, Shusuke Yagi, Daiju Fukuda, Hirotsugu Yamada, Takeshi Soeki, Tetsuzo Wakatsuki, Masataka Sata

**Affiliations:** 1 Department of Cardiovascular Medicine, Tokushima University Hospital, Tokushima 770-8503, Japan; yuuitirou_0110@yahoo.co.jp (Y.O.); pangtong2004@yahoo.ne.jp (R.Z.); isetaka@tokushima-u.ac.jp (T.I.); yamakoji3@tokushima-u.ac.jp (K.Y.); syagi@tokushima-u.ac.jp (S.Y.); daiju.fukuda@tokushima-u.ac.jp (D.F.); soeki@tokushima-u.ac.jp (T.S.); wakatsukitz@tokushima-u.ac.jp (T.W.); masataka.sata@tokushima-u.ac.jp (M.S.); 2 Clinical Research Center for Developmental Therapeutics, Tokushima University Hospital, Tokushima 770-8503, Japan; okayama0317@outlook.com; 3 Center for Cerebral and Cardiovascular Disease Information, National Cerebral and Cardiovascular Center, Osaka 564-8565, Japan; nakai.michikazu@ncvc.go.jp (M.N.); ysumi@ncvc.go.jp (Y.S.); 4 Department of Community Medicine for Cardiology, Tokushima University Graduate School of Biomedical Sciences, Tokushima 770-8503, Japan; yamadah@tokushima-u.ac.jp

**Keywords:** heart failure, echocardiography, mortality, big data

## Abstract

Background: Echocardiography requires a high degree of skill on the part of the examiner, and the skill may be more improved in larger volume centers. This study investigated trends and outcomes associated with the use and volume of echocardiographic exams from a real-world registry database of heart failure (HF) hospitalizations. Methods: This study was based on the Diagnosis Procedure Combination database in the Japanese Registry of All Cardiac and Vascular Datasets (JROAD-DPC). A first analysis was performed to assess the trend of echocardiographic examinations between 2012 and 2016. A secondary analysis was performed to assess whether echocardiographic use was associated with in-hospital mortality in 2015. Results: During this period, the use of echocardiography grew at an average annual rate of 6%. Patients with echocardiography had declining rates of hospital mortality, and these trends were associated with high hospitalization costs. In the 2015 sample, a total of 52,832 echocardiograms were examined, corresponding to 65.6% of all HF hospital admissions for that year. We found that the use and volume of echocardiography exams were associated with significantly lower odds of all-cause hospital mortality in heart failure (adjusted odds ratio (OR): 0.48 for use of echocardiography and 0.78 for the third tertile; both *p* < 0.001). Conclusions: The use of echocardiography was associated with decreased odds of hospital mortality in HF. The volumes of echocardiographic examinations were also associated with hospital mortality.

## 1. Introduction

Heart failure (HF) is a leading cause of cardiovascular death and remains a major socioeconomic issue [1,2]. Despite advances in modern therapy, mortality rates for HF continue to be high. Echocardiography is an easily available, versatile, and cost-effective means of cardiac imaging. This technique does not require intervention, radiation exposure, or radioactive isotopes [3]. It also provides data on cardiac dysfunction, hemodynamic status, valvular heart disease, and myocardial ischemia for treatment of heart failure [4]. There are several recommendations for the use of echocardiography in acute hospitalized HF [5,6]; however, clinical evidence and data on echocardiographic use are limited. Studies on nationwide data in the United States showed that, even if echocardiographic examinations were underused during cardiovascular hospitalizations, the use of echocardiography itself was associated with lower inpatient mortality [7]. On the other hand, echocardiography requires a high degree of skill on the part of the examiner, and skill may be more improved in large volume centers [8]. We hypothesized that higher hospital echocardiographic volumes would be associated with reduced inpatient mortality in patients admitted with acute heart failure. We analyzed trends and outcomes in association with the use and volumes of echocardiography using real-world big data based on HF hospitalizations to check the association between echocardiographic volumes and inpatient mortality.

## 2. Methods

### 2.1. Study Population 

First, we queried the Japanese Registry of All Cardiac and Vascular Diseases and the Diagnosis Procedure Combination (JROAD-DPC) database to quantify temporal trends in inpatient echocardiographic use between April 2012 and March 2017. Second, we explored the 2015 database to investigate whether the use and volume of echocardiographic exams were associated with all-cause in-hospital mortality in patients with HF. JROAD-DPC is a nationwide registry, a medical database with information on admission and discharge for cardiovascular diseases, clinical examinations and treatment status, patient status and hospital overview. The JROAD-DPC database integrates the information with analysis datasets covering 5.1 million cases between April 2012 and March 2017. The identification of HF (I50.0, I50.1, I50.9) hospitalization was based on the International Classification of Diseases (ICD) diagnosis codes. The diagnosis of HF was defined as the main diagnosis, admission-precipitating diagnosis, or most resource-consuming diagnosis. We defined readmission as an admission after discharge to a DPC hospital due to HF. Data regarding patient age and sex, main diagnosis, comorbidity at admission, length of hospitalization and treatment content were extracted from the database. We identified the numbers of board-certified doctors through the Japanese Circulation Society who were working full time in cardiovascular departments in Japan, and the numbers of transthoracic echocardiographies through the survey of the Japanese Circulation Society. The Institutional Review Board of the Tokushima University Hospital approved the study protocol (no. 3503).

**Figure 1:**
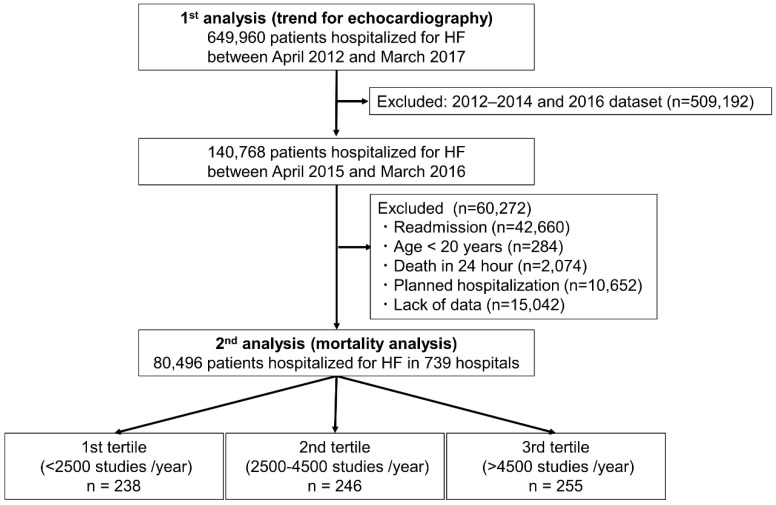
Flowchart of this study. HF, heart failure.

Figure 1 shows the patient selection flowchart. We selected 649,960 patients hospitalized with HF between April 2012 and March 2017. To analyze the associations between echocardiography and all-cause in-hospital mortality in HF, we enrolled 140,768 patients hospitalized for HF at 739 hospitals between April 2015 and March 2016. To reduce the variability of the data collected from this database, we focused on one year of data. We excluded patients from readmission cases (*n* = 42,660), age < 20 years (*n* = 284), death in 24 h after admission to exclude counter bias by patients in whom a fatal outcome occurred without sufficient lead time to obtain an echocardiographic examination if clinically indicated (*n* = 2074), a planned hospitalization to focus on hospital mortality (*n* = 10,652), and a lack of data (*n* = 15,042). As a result, a total of 80,496 were selected to assess hospital mortality. We categorized the 739 hospitals into 3 groups according to the number of echocardiographic studies performed annually (first tertile: <2500 cases, second tertile: 2500–4500 cases, third tertile: >4500 cases) and compared the groups of patients admitted to the categorized hospitals.

### 2.2. Clinical Outcomes

The main outcome was in-hospital mortality (total number of deaths during hospitalization).

### 2.3. Statistical Analysis 

Temporal trends in the incidence of echocardiographic use between 2012 and 2016 were analyzed. The increase in volume was analyzed by calculating the average annual percentage change from 2012 to 2016. The 2015 sample was dichotomized into three groups based on echocardiographic volumes, and descriptive statistics were generated on frequencies and proportions for categorical variables. Continuous variables are expressed as the mean ± SD for parameters with a normal distribution, as the median (interquartile range; IQR) for parameters with a skewed distribution, and categorical variables as the proportion (%). We estimated the odds ratios (ORs) and their 95% confidence interval (CI) for in-hospital mortality using multivariable models. A forward stepwise multivariable analysis was performed using relevant variables (associated with outcomes), incorporating a *p* value threshold <0.05 as the entry cutoff. All statistical tests were 2-sided, and *p* values less than 0.05 were considered statistically significant. Statistical analysis was performed using SAS version 9.4 and JMP 14.0 (SAS Institute Inc., Cary, NC). Odds ratios (ORs) and their 95% confidence interval (CI) for in-hospital mortality were calculated using multivariable models of multinomial logistic regression analysis in 3 groups.

## 3. Results

### 3.1. Analysis of Trends

**Figure 2:**
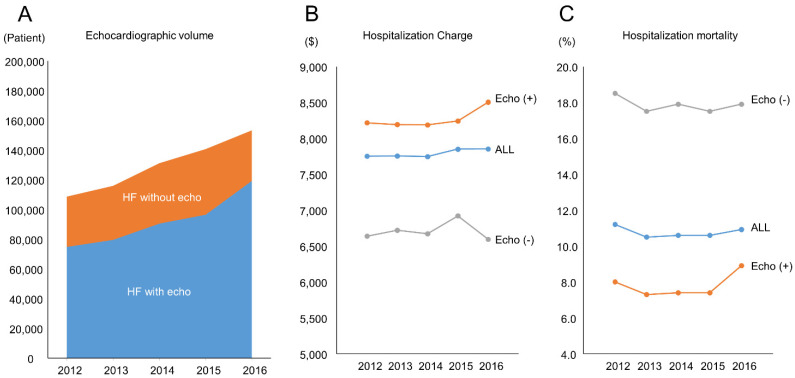
Trends in echocardiography use between 2012 and 2016. (**A**) Echocardiographic volume; (**B**) Hospitalization Charge; (**C**) Hospitalization mortality; HF, heart failure; $, US dollar.

A total of around 90,000 echocardiographic examinations per year were performed at all centers participating in this database from 2012 to 2016. During this period, the use of echocardiography grew at an average annual rate of 6% (Figure 2A). The numbers of in-hospital deaths were 12,206 (mortality: 11.2%), 12,159 (mortality: 10.5%), 13,959 (mortality: 10.6%), 14,895 (mortality: 10.6%), and 16,720 (mortality: 10.9%) in 2012, 2013, 2014, 2015 and 2016, respectively. An evaluation of resource use and patient outcomes found that patients with echocardiographic examinations had relatively lower rates of hospital mortality compared with patients without echocardiographic examinations. There was no trend of mortality among years. On the other hand, patients with echocardiographic examinations were associated with higher hospitalization costs than those without examinations (Figure 2B,C). According to these trends, we undertook a detailed analysis to investigate whether the use of echocardiography may be related to hospital outcomes.

### 3.2. 2015 Nationwide Inpatient Characteristics

Table 1 and Table S1 display the 2015 sample and the study population stratified by echocardiographic use and categorical variables. A total of 53.6% of patients in this study were male. The mean age was 78 ± 13 years, and half of all patients had hypertension (51.3%). Around 60% of the patients were New York Heart Association (NYHA) class III or IV. A total of 58,921 echocardiograms were examined in 2015, corresponding to 73.2% of all HF hospital admissions for that year. The distributions observed within categorical variables between the echocardiography and non-echocardiography groups of our study population are shown in Table 2. In a comparison of the two sample groups, patients undergoing echocardiography were younger, had a higher BMI, had a higher prevalence of hypertension dyslipidemia atrial fibrillation, and had less chronic kidney disease (all *p* < 0.001) (Table 2). The distribution of other comorbidities was comparable to the echocardiographic status.

### 3.3. Mortality Analysis

In the analysis cohort, in-hospital mortality was 7.7% (*n* = 6179). Patients with echocardiography had a significantly lower in-hospital mortality (6.0% vs. 12.2%, *p* < 0.001; OR, 0.46, 95% CI: 0.44–0.49). In the univariate logistic regression analysis, many clinical variables were associated with in-hospital mortality. A multivariate logistic regression adjusting for clinical variables (age, body mass index, NYHA class, hypertension, dyslipidemia, atrial fibrillation, stroke, renal failure, liver failure, cancer, artificial respirator, intra-aortic balloon pumping, percutaneous cardiopulmonary support, inotropes, percutaneous coronary intervention, board-certified doctor) using stepwise selection was performed to evaluate for the association between echocardiographic use and the odds of a patient’s death. To adjust by the number of doctors, we included board-certified doctors in this analysis. We found that the use of echocardiograms was associated with significantly lower odds of all-cause hospital mortality in heart failure (adjusted OR: 0.48; 95% CI: 0.45 to 0.51; *p* < 0.001) (Table 3).

**Figure 3:**
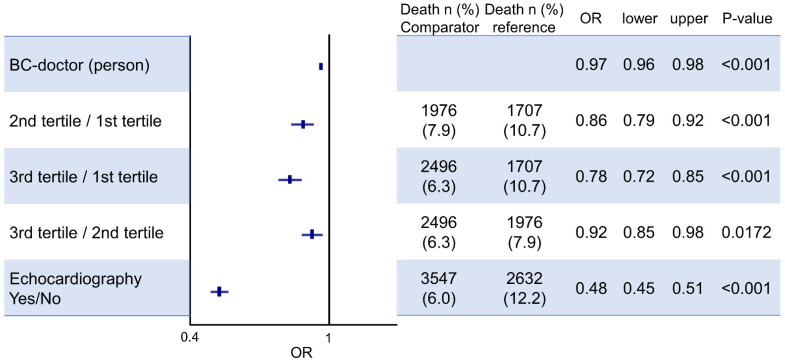
Odds ratio of in-hospital mortality. BC, board-certified. Dots and lines mean odds ratio (OR) and 95% CI, respectively.

In the tertile analysis of the number of echocardiographic volumes, the unadjusted OR for in-hospital mortality was significantly lower in the second tertile (OR, 0.72; 95% CI: 0.67–0.77, *p* < 0.001) and third tertile (OR, 0.56; 95% CI: 0.53–0.60, *p* < 0.001) than in the first tertile. In the stepwise analysis, the adjusted OR for in-hospital mortality was 0.86 (95% CI: 0.79–0.98, *p* < 0.001) for the second tertile and 0.78 (95% CI: 0.72–0.85, *p* < 0.001) for the third tertile. Interestingly, the number of echocardiographic examinations and volumes of the institute remained an independent risk factor after adjustment of clinical variables including the numbers of board-certified doctors (Figure 3).

## 4. Discussion

Echocardiography is common in many clinical aspects of cardiovascular hospitals. However, there are limited data for applications of echocardiography in Japan. Our main findings of this study were: (1) between 2012 and 2016, the use of echocardiography grew at an average annual rate of 6%, (2) patients with echocardiography had declining rates of hospital mortality and these trends were associated with high hospitalization costs, (3) HF patients with echocardiography in the hospitals with larger echocardiographic volumes had significantly lower in-hospital mortality, even after the matching of clinical variables, including the numbers of board-certified doctors.

### 4.1. Use of Echocardiogram on HF

In the United States, the analysis of the largest publicly available all-payer impatient database showed that the incidence of echocardiography per hospitalization gradually increased at annual rates of around 3.0% [7]. However, their analysis suggested that echocardiography may be underused for common and appropriate indications because the incidence of echocardiographic performance per hospitalization was around 2% in 2010. In Japan, 58,921 echocardiograms were examined in 2015, corresponding to 73.2% of all HF hospital admissions for that year, and the incidence of echocardiography is extremely high compared with the United States. This discrepancy reflects differences in study populations because our demographics were specific to the HF inpatient setting and there were high rates of echocardiographic use among cardiovascular hospitals in Japan.

### 4.2. Impact of Echocardiograhy on HF Mortality 

In 2015, a total of 58,921 echocardiograms were examined in HF. After adjusting for key variables, we observed that the use of echocardiography was associated with lower odds of hospital mortality in this cohort. Interestingly, the total number of exams performed at institutes was strongly associated with hospital mortality even after the adjustment of the numbers of board-certified doctors. In a previous study for acute coronary syndrome, patients who were admitted to cardiology centers more commonly underwent cardiac catheterization, received evidence-based therapy and had a significantly lower mortality than those admitted to noncardiology centers [9]. Some studies also showed that a large operating volume, number of cardiologists and cardiovascular beds were associated with reduced mortality [10,11]. The similar causal link between examination totals and mortality may be true for echocardiography. Echocardiography is a first-line diagnostic tool that contributes to the initiation of therapy in HF [5,6]. It was well known that echocardiographic decisions improved mortality rates in the clinical setting [12]. In a major academic medical center, 32% of inpatient echocardiographic examinations led to an active change in medical care including cardiovascular managements [13]. The clinical information provided by echocardiography can assist physicians in management decisions and patient risk stratification. Another explanation may show that in large university teaching hospitals with higher resources and advanced procedures in patients with HF, the usage of echocardiography should be higher than in smaller institutions, and the better outcome of patients in the first ones might be explained by easier access to echocardiography and an earlier diagnosis of change in the heart function or of complications, which can lead to a better outcome. In addition, improvement in HF outcomes over time may be attributable to an increasing use of guideline-directed medical therapy, new therapies (such as sacubitril/valsartan), a shift towards more HF with preserved EF and less HF with reduced EF, increasing use of mechanical support, etc. All of these things may be more common in larger centers that do more echoes. These scenarios may support our hypothesis that the use of echocardiography can improve the hospital mortality in HF. According to our results, the quality of care including echocardiography may be associated with outcomes in patients with HF. Because some studies showed that quality of care for patients with HF may be sub-standard and that there is a wide heterogeneity in the quality of care for HF among hospitals in several countries [14,15], the quality of care for patients should be improved in the future. Among patients with HF, in-hospital mortality is reported to vary from around 5.0% to 12.0% [16,17,18,19]. Our cohort had a mortality rate of about 10%, which may reflect the current situation in Japan as a hyper-aged society.

### 4.3. Clinical Implication 

Improvement in the prognosis of HF is an essential part of clinical management [5]. According to our data from the large high-risk HF cohort, including around 60% of patients with NYHA III/IV, patients with echocardiography had a lower mortality after the adjustment for clinical variables, including the numbers of board-certified doctors. This study suggests that improving the quality of echocardiographic examinations could be beneficial and that the skill of echocardiography contributes to patients’ care and prognosis.

### 4.4. Limitations

The study based on ICD codes has several limitations. We analyzed only patients with HF hospitalized in facilities contributing to the database, which may lead to selection bias. This database included approximately 50% of Japanese Circulation Society certified hospitals and 29% of all hospitals in Japan [20]. All hospitals were cardiovascular training facilities and their affiliated facilities. We were unable to gather the data from noncardiology wards (e.g., internists or geriatric wards). The database has no information on laboratory data (e.g., NT-proBNP) and specific echocardiographic data (e.g., left ventricular ejection fraction) to assess the prognosis of HF. To overcome this issue, we used treatment devices and isotropic medication as markers of HF severity. All-cause mortality was used as the primary end point in our patient population. The most likely cause of death in our patient population is HF, given the known high-risk nature of our patient population. The patients in this study are mostly Japanese. The results may differ due to racial/cultural differences in other countries. The JROAD-DPC dataset extracts only a record, which contains all types of cardiovascular diseases in any categories of diagnosis based on the DPC dataset in the Ministry of Health, Labor and Welfare in Japan. This J-ROAD dataset has already been validated in past studies [21,22]. Around 40% of acute HF patients had NYHA I–II symptoms in this database. Many patients with even mild symptoms were admitted to hospitals with board-certified doctors, probably due to the insurance system that covers all of its citizens in Japan. In clinical practice, there are a subset of patients less likely to be imaged or to receive aggressive care, in part because it is understood that overall outcomes will be poor. We focused on the 2015 database because the data before 2015 had some missing values including the NYHA functional class. The short period of inclusion was another limitation. Finally, we have excluded almost 40% of the entire cohort for various reasons, and thus there is a selection bias. According to these limitations, this paper should be considered as a hypothesis-generating study.

## 5. Conclusions

The use of echocardiography was associated with decreased odds of hospital mortality in HF. The number of echocardiographic examinations was also associated with hospital mortality; thus, this study could generate the hypothesis that the skill of echocardiography may be better in the large volume centers and contribute to good outcomes in HF. Our study is based on a highly and retrospectively selected cohort. We believe that larger international studies are warranted to confirm this result.

## Figures and Tables

**Table 1:** Baseline hospital characteristics.

Hospital Characteristics	All	1st Tertile	2nd Tertile	3rd Tertile
Number of Institutes	739	238	246	255
BC Doctor	4 (2–6)	2 (1–3)	4 (3–5)	7 (4–10)
TTE	3315 (2070–5361)	1733 (1220–2043)	3294 (2888–3943)	6613 (5293–8543)
Bed	386 (275–536)	279 (198–342)	394 (301–487)	560 (405–701)
CV Bed	35 (28–44)	30 (20–37)	33 (30–42)	41 (33–51)

Data are presented as the median (interquartile range). Abbreviations: BC, board-certified; CV, cardiovascular.

**Table 2:** Characteristics with and without echocardiography.

	All	Echocardiography (−)	Echocardiography (+)	*p*-Value
N	80,496	21,575	58,921	
Age (year)	78 ± 13	78.4 ± 12.5	77.8 ± 12.7	<0.001
Male (%)	53.6	54.3	53.4	0.0201
BMI (%)	22.8 ± 5.2	22.7 ± 5.4	22.8 ± 5.1	<0.001
NYHA I-II (%)	41.4	43.9	40.5	<0.001
Complication (%)				
HT	51.3	49.3	52.0	<0.001
DM	26.8	26.8	26.8	0.963
DL	17.6	16.5	18.0	<0.001
MI	9.8	9.7	9.8	0.7042
Af	34.3	30.1	35.9	<0.001
PVD	3.9	4.0	3.8	0.1486
Stroke	7.8	8.0	7.7	0.1892
Dementia	5.5	5.6	5.4	0.3819
COPD	7.0	6.9	7.1	0.3131
Liver Disease	0.1	0.2	0.1	0.1098
CKD	14.5	17.0	13.5	<0.001
Cancer	10.8	10.3	11.0	0.0029
Treatment (%)				
HD	5.0	7.1	4.2	<0.001
Artificial Respirator	19.3	22.2	18.2	<0.001
IABP	0.9	0.7	1.0	<0.001
PCPS	0.1	0.2	0.1	0.0271
Inotropes	12.0	11.8	12.0	0.364
PCI	4.7	3.3	5.3	<0.001

Data given as the proportion. See abbreviations in Table 1. Abbreviations: OR, odds ratio.

**Table 3:** Multivariate analysis of covariates for in-hospital mortality in all patients.

	Univariate				Multivariate			
	OR	Lower	Upper	*p*	OR	Lower	Upper	*p*
Age	1.06	1.06	1.07	<0.001	1.07	1.07	1.07	<0.001
BMI	0.90	0.89	0.91	<0.001	0.95	0.94	0.96	<0.001
Male	1.29	1.23	1.36	<0.001				
NYHA 1–2	1.86	1.76	1.97	<0.001	0.67	0.63	0.71	<0.001
HT	0.35	0.33	0.37	<0.001	0.43	0.40	0.45	<0.001
DM	0.73	0.69	0.78	<0.001				
DL	0.34	0.31	0.38	<0.001	0.56	0.51	0.63	<0.001
MI	1.06	0.97	1.16	0.1743				
Af	0.65	0.61	0.69	<0.001	0.74	0.69	0.79	<0.001
PVD	0.98	0.86	1.13	0.8059				
Stroke	1.77	1.63	1.92	<0.001	1.44	1.32	1.58	<0.001
Dementia	1.87	1.7	2.05	<0.001				
COPD	1.03	0.93	1.14	0.5749				
CKD	1.42	1.33	1.52	<0.001	1.36	1.26	1.47	<0.001
Liver Failure	2.94	1.83	4.75	<0.001	3.14	1.79	5.52	<0.001
Cancer	1.48	1.38	1.60	<0.001	1.52	1.40	1.65	<0.001
HD	1.3	1.17	1.45	<0.001				
Artificial Respirator	3.29	3.12	3.48	<0.001	2.74	2.57	2.93	<0.001
IABP	3.86	3.25	4.58	<0.001	1.46	1.16	1.83	0.0011
PCPS	19.34	13.25	28.23	<0.001	12.19	7.53	19.74	<0.001
Inotropes	6.46	6.11	6.84	<0.001	5.75	5.37	6.14	<0.001
PCI	0.46	0.38	0.54	<0.001	0.24	0.20	0.29	<0.001
TTE	0.46	0.44	0.49	<0.001	0.48	0.45	0.51	<0.001
All Beds	1.00	1.00	1.00	<0.001				
CV Beds	0.99	0.99	0.99	<0.001				
BC Doctor	0.95	0.94	0.95	<0.001	0.97	0.96	0.98	<0.001
Group 1st Tertile vs 2nd	0.72	0.67	0.77	<0.001	0.86	0.79	0.92	<0.001
2nd vs 3rd	0.56	0.53	0.60	<0.001	0.78	0.72	0.85	<0.001
2nd vs 3rd	0.78	0.74	0.83	<0.001	0.92	0.85	0.98	0.0172

Data given as the proportion. See abbreviations in Table 1. Abbreviations: OR, odds ratio.

## Data Availability

Individual anonymized data supporting the analyses contained in the manuscript will be made available upon reasonable written request from researchers whose proposed use of the data for a specific purpose has been approved.

## Supplementary Materials

The following are available online, Table S1: Baseline patient characteristics.

## Abbreviations

HF = heart failure, DPC = Diagnosis Procedure Combination, ICD = International Classification of Diseases, NYHA = New York Heart Association.
