# Supplementary material for: Use of Echocardiography and Heart Failure In-Hospital Mortality from Registry Data in Japan
Source: J Cardiovasc Dev Dis. 2021 Sep 30;8(10):124. doi: 10.3390/jcdd8100124 (PMC8536984; doi:10.3390/jcdd8100124)
Supplement: Supplementary file 1 [file jcdd-08-00124-s001.zip › jcdd-1355599-supplementary.pdf]

Supplemental Table S1: Baseline patient characteristics.

	All	1st tertile	2nd tertile	3rd tertile
Number or patients	80,496	15,975	24,991	39,530
Age (year)	78±13	81±12	78±13	77±13
Male (%)	53.6	49.4	53.4	55.5
BMI	22.8±5.2	22.5±4.9	22.8±4.7	22.8±5.6
NYHA I-II (%)	41.4	41	39.5	42.8
Complication (%)				
HT	51.3	46.9	53.0	52.0
DM	26.8	25.1	27.2	27.2
DL	17.6	12.1	16.9	20.3
MI	9.8	8.2	9.7	10.5
Af	34.3	33.8	35.0	34.2
PVD	3.9	3.4	3.7	4.2
Stroke	7.8	10.5	8.1	6.5
Dementia	5.5	7.1	5.7	4.7
COPD	7.0	8.6	7.0	6.5
Liver disease	0.1	0.2	0.1	0.1
CKD	14.5	14.3	14.2	14.7
Cancer	10.8	11.3	10.8	10.6
Treatment (%)				
HD	5.0	4.3	4.7	5.5
Artificial respirator	19.3	13.4	18.8	22
IABP	0.9	0.4	0.6	1.3
PCPS	0.1	0.02	0.1	0.2
Inotropes	12.0	12.4	11.6	12.1
PCI	4.7	3.5	4.8	5.2
TTE	73.2	72.1	72.7	73.9

Data are presented as percentage of patients. Abbreviations; BMI, body mass index; NYHA, New York heart association functional class; HT, hypertension, DM, diabetes mellitus, DL, dyslipidemia, MI, myocardial infarction; Af, atrial fibrillation; PVD, peripheral vascular disease; COPD, chronic obstructive pulmonary disease; CKD,

chronic kidney disease; HD, hemodialysis, IABP, intra-aortic balloon pumping; PCPS, percutaneous cardiopulmonary system; PCI, percutaneous coronary intervention; TTE, transthoracic echocardiogram.
